# Being Strange While Being No One

**DOI:** 10.3389/fpsyg.2018.00500

**Published:** 2018-04-09

**Authors:** Ulrike S. Pompe-Alama

**Affiliations:** Institute for Philosophy, University of Stuttgart, Stuttgart, Germany

**Keywords:** self-model theory, personality disorders, being no one, therapy, mental content

In *Being No One*, Thomas Metzinger might have—unknowingly—opened a gate to the (neuro-)philosophical investigation into Personality Disorders (PD) (Metzinger, 2004). The following is an outline of the merits of the Self-Model Theory (SMT) for psychiatric research. The here-proposed roadmap goes beyond Metzinger's own vision, but is rooted in and inspired by the framework developed in *Being No One*.

According to the DSM-5, a personality disorder “is an enduring pattern of inner experience and behavior that deviates markedly from the expectations of the individual's culture, is pervasive and inflexible, has an onset in adolescence or early adulthood, is stable over time, and leads to distress or impairment” (American Psychiatric Association, 2013).

Today, the different forms of PD are distinguished by predominant traits such as paranoia, excessive emotionality, lack of empathy, heightened egocentricity, grandiosity, etc., but not by a diagnosis of an underlying neurological impairment. The description remains on the behavioral level, sparing mechanisms, and origins. Patients are locked in a pattern of self-reinforcing and mutually consolidating emotions and cognitive states. The diagnosis represents a holistic attribution, addressing not a single cognitive or emotional impairment, but the overall personality style. Pinpointing the underlying neuropsychological deficit is difficult. In most discussions of PD, e.g., pathological narcissism, it becomes apparent that, among other things, emotional and affective self-regulation is impaired and that many behavioral and cognitive outputs serve to alleviate the emotional distress. We thus face a behaviorally complex phenomenon, which encompasses a wide variety of interdependent components of the cognitive system, but up to today, we fail to understand the exact neuropsychological mechanisms behind these patterns.

Metzinger's SMT as developed in *Being No One* can help us to develop a comprehensive account of the first-person perspective of an afflicted person and explain the pervasiveness of the behavior. It furthermore invites a deepened discussion on the scope and limits of therapeutic intervention and the concept of mental health.

The concept of *personality* invokes the idea of a person's “true self.” Metzinger, however rejects the idea of the existence of “selves”—instead, what exists are merely the contents of a transparent Phenomenal Self Model (hereafter, PSM, cf. p. 626). This transparent PSM harbors various subpersonal factors of the mind, e.g., bodily states, emotional and affective experiences, and thoughts. It integrates what Metzinger calls “self-representational content” (p. 302), which includes self-referential contents as well as social and intersubjective contents. In addition to these so-called transparent contents, Metzinger posits opaque mental contents; whereas the transparent components are experienced as “directly and immediately given” (p. 400), the opaque component “is often but not necessarily experienced as deliberately constructed, for instance, as one's own thought” (p. 400). The specific subjective perspective—how it feels to be someone—is created by bundling the different input components through a feeling of “mineness.” Metzinger refers to this as a *simulation* of a self.

The concept of personality, however, is not obsolete, as shown by the discussion of Dissociative Identity Disorder (DID). Metzinger refers here to the concept of personality in the sense of a “currently active self-model” (p. 528).

DID is understood to be a splitting of the phenomenal self under intense emotional pressure. The SMT is able to provide an explanation because the phenomenon of DID “shows how psychological properties standardly ascribed to the system as a whole on the personal level of description are determined by the subpersonal processes which generate the content and the functional properties of the currently active self-model. Identity disorders, while being diagnosed on the *personal* level of description, result from subpersonal disintegration” (p. 528). The same line of reasoning can hold for PD. As Metzinger states: “The concept of PSM forms the logical link between subpersonal and personal levels of description. As a neurobiological, functional, and representational entity it is subpersonal, but by satisfying the transparency constraint […], it generates personal-level properties like phenomenal selfhood for the system as a whole, enabling “strong” first-person phenomena, social cognition, and intersubjectivity” (p. 528). In PD, we are dealing with exactly the last three issues: a markedly different first person-perspective, impaired and deviant social cognition and impaired or even lacking intersubjectivity. If personality styles, i.e., the observable and characteristic behavioral pattern which is stable over time, result from misinforming subpersonal level information channels, we should consider analyzing PD along these lines and ask how the transparent components of a PD afflicted person's PSM give rise to opaque contents. Thus, we need to consider the subpersonal and transparent emotional and affective set-up which drives the acute phenomenal experiences of the patient (e.g., an acute sense of prosecution) and analyze the ensuing cognitive attribution toward the phenomenal self, which seeks to rationalize and objectify the felt state, creating explanatory thoughts at the personal level, which drive and consolidate the behavioral pattern. As an example, consider again the case of pathological narcissism where self-aggrandizing thoughts result from an acute need to defend the self against self-defeating affective states, like an acute feeling of emptiness or worthlessness.

Does SMT open new avenues for therapeutic intervention? Yes and No.

On the one hand, Metzinger states that “phenomenal selfhood originates in a lack of attentional, sub-symbolic self-knowledge. Phenomenal transparency is a special kind of darkness” (p. 632). If this is true, then bringing subsymbolic contents to the surface for rational scrutiny might be impossible. On the other hand, in his discussions of future applications of the SMT, Metzinger hopes for a kind of personality enhancement by “functionally integrating cognitive insight, emotional self-modeling, and actual behavioral profile” (p. 631 ff.) in order to “maximize the internal coherence of the self-model” (p. 632). As Metzinger concedes, the consistency and coherence constraint can shape our understanding of what it means to be mentally healthy (cf. p. 632). In PD, where violating social norms and lacking “moral integrity” (p. 632) are given, therapeutic intervention might thus target the PSM.

The SMT allows us to not only model the first-person perspective of a PD-afflicted mind, we can also access the underlying factors by splitting the overall phenomenon into transparent and opaque contents of the various self-models, thereby making an intricate and complex psychiatric riddle a bit more manageable. Metzinger himself formulates the explanatory power of the SMT as follows: “The phenomenally available content of the self-model is an excellent constraint to differentiate between different phases of childhood development, certain nosological stages in psychiatric diseases, or the unfolding of social competence in the animal kingdom” (p. 304). All three aspects—social competence, developmental aspects, and psychiatry—play a role in PD. All we need to do is fill in the blanks.

## Author contributions

The author confirms being the sole contributor of this work and approved it for publication.

### Conflict of interest statement

The author declares that the research was conducted in the absence of any commercial or financial relationships that could be construed as a potential conflict of interest.
